# Impact of a syndrome-specific antibiotic stewardship intervention on antipseudomonal antibiotic use in inpatient diabetic foot infection management

**DOI:** 10.1017/ash.2023.123

**Published:** 2023-03-02

**Authors:** Randy J. McCreery, Elizabeth Lyden, Matthew Anderson, Trevor C. Van Schooneveld

**Affiliations:** 1 Department of Internal Medicine, Section of Infectious Diseases, University of Nebraska Medical Center, Omaha, Nebraska; 2 College of Public Health, University of Nebraska Medical Center, Omaha, Nebraska

## Abstract

**Objective:**

To demonstrate that a syndromic stewardship intervention can safely reduce antipseudomonal antibiotic use in the treatment of inpatient diabetic foot infections (DFIs).

**Intervention and method:**

From November 2017 through March 2018, we performed an antimicrobial stewardship intervention that included creation of a DFI best-practice guideline, implementation of an electronic medical record order set, and targeted education of key providers. We conducted a retrospective before-and-after study evaluating guideline adherent antipseudomonal antibiotic use 1 year before and after the intervention using interrupted time-series analysis.

**Setting:**

University of Nebraska Medical Center, a 718-bed academic medical center in Omaha, Nebraska.

**Patients:**

The study included 193 adults aged ≥19 years (105 in the preintervention group and 88 in the postintervention group) admitted to non–intensive care units whose primary reason for antibiotic treatment was diabetic foot infection (DFI).

**Results:**

Guideline-adherent use of antipseudomonal antibiotics increased from 39% before the intervention to 68% after the intervention (P ≤ .0001). Antipseudomonal antibiotic use decreased from 538 days of therapy (DOT) per 1,000 DFI patient days (PD) before the intervention to 272 DOT per 1,000 DFI PD after the intervention (P < .0001), with a statistically significant decrease in both level of use and slope of change. We did not detect any changes in length of stay, readmission, amputation rate, subsequent positive Clostridioides difficile testing, or mortality.

**Conclusions:**

Our 3-component intervention of guideline creation, implementation of an order set, and targeted education was associated with a significant decrease in antipseudomonal antibiotic use in the management of inpatient DFIs. DFIs are common and should be considered as opportunities for syndromic stewardship intervention.

Antimicrobial stewardship programs (ASPs) are an integral part of the United States public health strategy to combat the emergence of antimicrobial resistant bacteria.^1^ Syndromic stewardship interventions have been used by ASPs to reduce unnecessary antibiotic use and to improve patient care in common infections.^2,3^ In 2010, 77,491 hospital discharges were attributed to diabetic foot infections (DFIs), making this a common hospital-managed infection. However, published strategies to improve antibiotic use in these cases are lacking.^2–4^

National guidelines for the treatment of DFI were published in 2012 and include antibiotic recommendations based upon severity and prior culture data.^5^ A few reports have suggested that adherence to antibiotic prescribing guidelines in DFI is suboptimal, but when guidelines are followed, improved outcomes have been observed.^6–9^ Clinical decision support systems (CDSSs) have been utilized with the goal of improving guideline-adherent treatment for infectious diseases, although mixed success has been reported.^10^ In DFI, CDSS associated with guideline adherence and reduction in unnecessary antibiotic use has not been reported.

A review of DFIs at our institution (July 2014–June 2015) revealed that *Pseudomonas aeruginosa* was an uncommon DFI pathogen, present in 1 (2%) of 56 cases, but DFI was empirically treated in 41 (73%) of 56 cases.^11^ This finding suggested that DFI was an excellent opportunity for a syndromic stewardship intervention. Between November 2017 and March 2018, we conducted a 3-part ASP intervention that included provider education and the creation of an institutional management guideline and DFI order set in the electronic medical record (EMR). We evaluated the effect of this intervention (education, guideline, and order set) on DFI antibiotic use and clinical outcomes.

## Methods

### Study setting

The University of Nebraska Medical Center (UNMC) is a 718-bed, academic medical center located in Omaha, Nebraska. Institution-specific guidelines for the treatment of common infectious diseases with associated EMR order sets (ie, for urinary tract infections, pneumonia, etc) has been a core strategy of the program, coupled with audit and feedback.

### Study design and population

We retrospectively assessed empiric antibiotic use in hospitalized patients with DFI before and after a stewardship intervention. Guideline-adherent antibiotic use was measured before the intervention (November 1, 2016–October 31, 2017) and was compared to guideline-adherent antibiotic use after the intervention (April 1, 2018–March 31, 2019), excluding an implementation period (November 1, 2017,–March 31, 2018). We included adults aged ≥19 years (19 is the age of majority in Nebraska) who had been admitted to general medical wards through internal medicine (IM) or family medicine (FM) practices and received antibiotic treatment for a DFI. The IM and FM services include hospitalist teams and comprise >90% of DFI admissions. We identified eligible cases using *International Classification of Diseases Tenth Revision* (ICD-10) codes for DFI. Code E8-E13 identifies patients with diabetes mellitus and among these, they must have had at least 1 of the following codes: 0J, 0Q, 0S, 0Y for procedures involving lower extremity tissue, bones or joints or A48.0, I96, L02, L03, L97, M86 Z44, Z89 identifying gangrene, cellulitis, abscess, ulcer, osteomyelitis, or limb loss. Code definitions are included in the Supplementary Material. Cases were manually reviewed, and only cases in which infection was described as originating in the foot were included. Infections originating at the ankle and above were excluded, as were surgical wound infections. If an infection was present or suspected at any other site not contiguous with the foot, the case was excluded. Additional exclusion criteria included intensive care unit (ICU) admission, transfer from an outside hospital, admission to non-IM/FM or hospitalist services (surgical services rarely admit these patients at our institution), puncture wound or foreign body present, or admitted from hospice. Only the first admission for DFI was included. All patients were unique.

## Study definitions

The presence of a DFI and infection severity were defined based on national guidelines.^5^ Infection was defined as having a wound present with at least 2 of the following: swelling, erythema, tenderness, warmth, or purulence. Severe infection was defined as an infection plus ≥2 of the following within 8 hours of admission: fever or hypothermia (temperature >38.0°C or <36.0°C), heart rate >90 beats per minute, respiratory rate >20 breaths per minute, or white blood cell count (WBC) >12,000/µL. Infections without evidence of systemic inflammatory response were consider nonsevere. Empiric coverage for *P. aeruginosa* was considered guideline adherent only if risk factors for *P. aeruginosa* were present (eg, water exposure or previous isolation as outlined in our institutional guideline). Empiric coverage was defined as the first systemic antibiotic regimen received. Topical antibiotics were excluded. Antibiotic use was measured in days of therapy (DOT), whereas any dose of an antibiotic in a single day constituted 1 day of therapy. Formulary antipseudomonal antibiotics included meropenem, piperacillin/tazobactam, cefepime, ceftazidime, aztreonam, levofloxacin, ceftolozane-tazobactam, ceftazidime-avibactam, and aminoglycosides. Only deep-tissue cultures were evaluated; they were categorized as having been obtained via surgical debridement (operative or bedside), amputation, or bone biopsy. All other wound cultures were excluded.

### Intervention

In July 2017, we produced an institutional guideline for the management of DFIs modeled after national guidelines and supplemented by local microbiologic data and formulary preferences (Appendix A).^5^ This guideline was reviewed by our local ASP committee and was made available online. Recommendations included defining severity and risk factors for resistant pathogens, such as MRSA and *P. aeruginosa*, with empiric antibiotic recommendations based upon these factors. Additionally, a comprehensive DFI admission order set was created in the EMR with severity- and risk-factor–stratified empiric antibiotic orders available. The order set included options for additional consultation (eg, surgical, infectious diseases, endocrinology, nephrology, etc), suggestions for appropriate imaging and laboratory tests, as well as options for other medications including insulin.

Between November 2017 and March 2018, education was provided to clinicians who commonly prescribe antibiotics in DFIs. Emergency medicine residents received 1 session, hospitalists including advanced practice providers received 1 session, internal medicine residents received 1 session, and family medicine residents and attending physicians received 2 sessions. Content included education on local microbiology highlighting the rarity of *P. aeruginosa* as a DFI pathogen, current prescribing data noting opportunities for improvement, and an introduction to our institutional guideline and order set outlining recommended antibiotic regimens. Education was conducted at either department meetings or educational conferences typically lasting 15–20 minutes (5 sessions total). Our ASP conducts audit and feedback but did not specifically target DFI for review. The UNMC Institutional Review Board classified this work as a quality improvement project.

### Data collection

We collected demographic, clinical, laboratory, microbiologic, and treatment data. EMR order-set use was tabulated via request from our local EMR team. Online guideline document downloads were collected using local information technology resources. For vital signs, maximum or minimum values along with maximum WBCs within 8 hours of admission were recorded. To calculate the Elixhauser comorbidity score for each patient, ICD-10 codes from the source encounter file were parsed using SAS codes. If any of the predetermined ICD codes for a particular condition existed for a patient in the source file, that patient was said to have that condition and was assigned the standard Elixhauser score for that condition. All Elixhauser scores for each condition were summed to yield the final Elixhauser comorbidity index for each patient.

Conditions that make up the Elixhauser comorbidity index included peripheral vascular disorders, depression, lymphoma, metastatic cancer, solid tumor without metastasis, renal failure, and congestive heart failure.

### Study outcomes

The primary end point of interest in this study was adherence to empiric antipseudomonal antibiotic use guidelines. Secondary outcomes included antipseudomonal antibiotic use measured in DOT per 1,000 DFI patient days (PD), length of stay (LOS), 30-day readmission, amputation, subsequent positive *Clostridioides difficile* testing within 30 days of admission, 30-day mortality, and 1-year mortality.

### Statistical analysis

Descriptive statistics including means, standard deviations, medians, minimums, maximums, counts, and percentages were used to summarize the data. Categorical data were compared between the 2 periods using the Fisher exact test. The independent sample *t* test or Mann-Whitney test was used to compare continuous data.

A negative binomial regression model for interrupted time series was used to compare the change in adherence rates prior to and after the intervention. We hypothesized that the intervention was associated with both a level change and a slope change in the rate of antipseudomonal therapy prescribed between the 2 periods following the approach outlined by Bernal et al.^12^

## Results

**Fig. 1.**
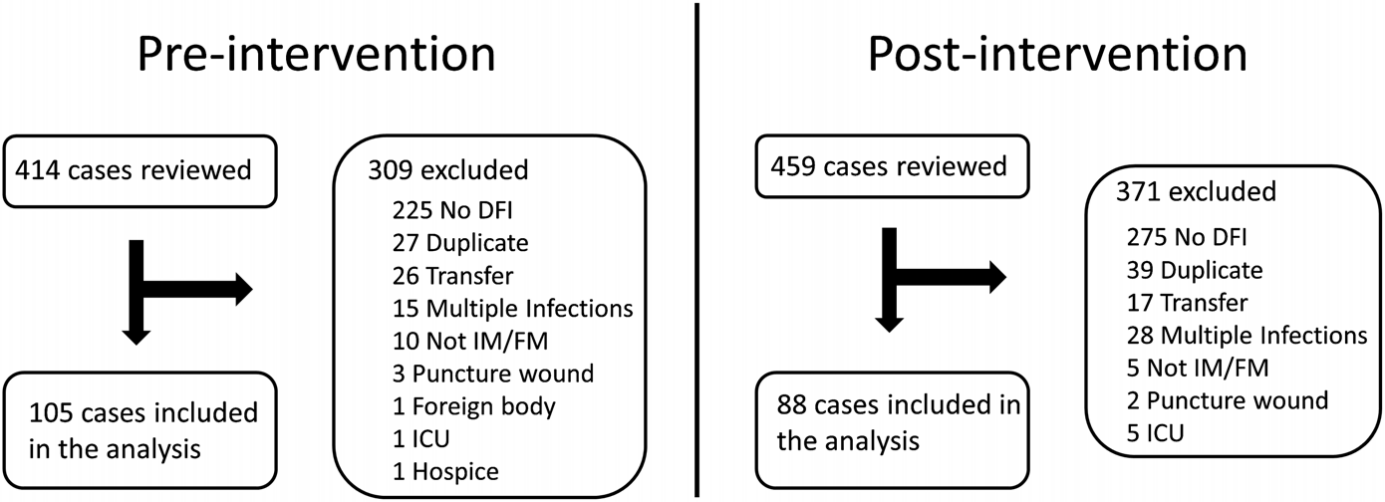
Study patient flow. Note. DFI, diabetic foot infection; IM, internal medicine; FM, family medicine; ICU, intensive care unit.

In total, 874 cases were identified and reviewed, and 681 were excluded (Fig. 1). We included 193 unique patients in the analysis: 105 in the preintervention period and 88 in the postintervention period.

Demographics and clinical characteristics are shown in Table 1. The groups were similar with respect to demographics, infection severity, and comorbidities. Clinical outcomes, including mortality, readmission, *Clostridioides difficile* infection (CDI) rates, LOS, and amputation rate, were not significantly different between the 2 periods (Table 2).

**Table 1.** Baseline Patient Demographic and Clinical Characteristics

Characteristic	Preintervention Period (N=105), No. (%)^a^	Postintervention Period (N=88), No. (%)^a^	*P* Value
Age, mean y (SD)	57.6 (12.6)	57.7 (11.7)	.95
Sex	61 (58.1)	59 (67.0)	.23
BMI, mean (SD)	35.0 (8.7)	34.1 (11.1)	.55
**Ethnicity**			
White	74 (70.4)	64 (72.7)	.66
Black	18 (17.1)	11 (12.5)	
Hispanic or Latino	10 (9.5)	8 (9.0)	
Other	3 (2.8)	5 (5.6)	
HbA1C, mean (SD)	9.1 (2.5)	9.2 (2.4)	.87
Heart rate, mean (SD)	95.1 (15.2)	95.1 (16.1)	.99
Respiratory rate, mean (SD)	19.6 (2.0)	19.7 (2.0)	.77
WBC, mean (SD)	11.5 (4.7)	12.1 (5.9)	.45
Systolic BP, mean (SD)	126.4 (21.4)	127.4 (20.2)	.73
Fever or hypothermia	27 (25.7)	27 (30.6)	.52
Elixhauser comorbidity index, mean (SD)	4.4 (5.5)	4.0 (4.9)	.67
Severe DFI	50 (47.6)	45 (51.1)	.66

Note. SD, standard deviation; no., number; BP, blood pressure; HbA1C, hemoglobin A1C; WBC, white blood cell count; DFI, diabetic foot infection.

a Units unless otherwise specified.

**Table 2.** Clinical Outcomes

Outcome	Preintervention Period (N=105), No. (%)^a^	Postintervention Period (N=88), No. (%)^a^	*P* Value
Guideline adherent empiric antipseudomonal antibiotic use	41 (39)	60 (68)	<.0001
Empiric antipseudomonal antibiotic use no. (%)	68 (64.7)	35 (39.7)	.0008
30-day mortality^b^	1 (0.9)	1 (1.2)	1.0
1-year mortality^b^	10.4 (10/96)	11.1 (6/54)	1.0
30-day readmission	18 (17.1)	18 (20.4)	.58
*C. difficile* within 30 d (n/N)	0 (0/105)	2.2 (2/88)	.20
LOS median d, (min–max)	4.9 (0.91–61)	5.1 (0.21–49)	.84
Amputation during admission	36 (34.2)	25 (28.4)	.43

Note. *C. difficile*, *Clostridioides difficile*; LOS, length of stay.

a Units unless otherwise specified.

b Mortality data were unavailable for 3 patients in the 30-day preintervention group and for 9 patients in the 1-year preintervention group. Mortality data were unavailable for 6 patients in the 30-day postintervention group and for 34 patients in the 1-year postintervention group.

**Fig. 2.**
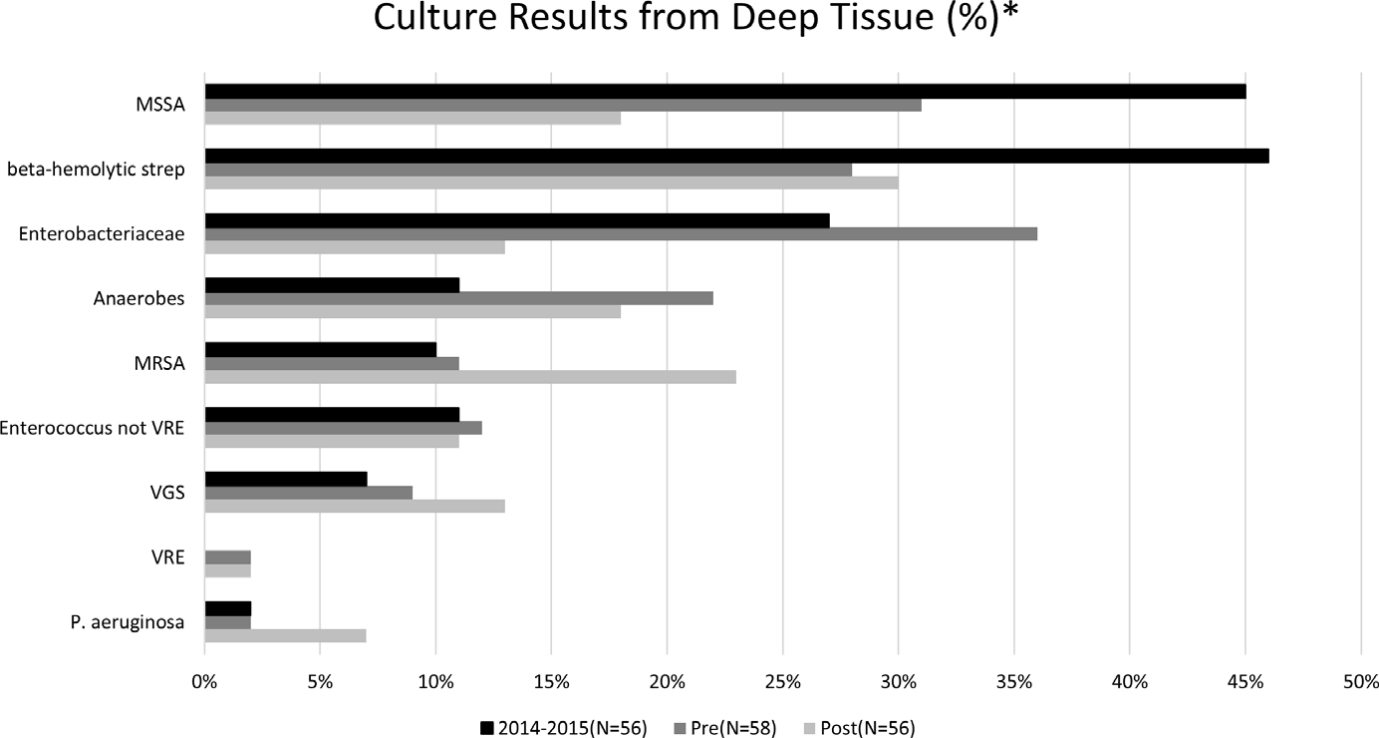
Deep-tissue culture results from 3 periods. *Chart displays the percent of cases with deep-tissue culture where a specific organism was present. Often >1 isolate per case. Note. MSSA, methicillin-susceptible *Staphylococcus aureus*; strep, streptococci; MRSA, methicillin-resistant *Staphylococcus aureus*; VRE, vancomycin-resistant *Enterococcus*; VGS, *viridans* group streptococci; P. aeruginosa, *Pseudomonas aeruginosa*.

Figure 2 presents deep-tissue culture results for 3 different periods: the preintervention period, the postintervention period, and the preliminary 2014–2015 DFI case-review period briefly described in the introduction. Of 170 cases with data, *P. aeruginosa* was isolated in 3.5% of cases.

A decrease in the rate of empiric antipseudomonal antibiotic use was observed in the postintervention group: 68 of 105 (64.7%) in the preintervention period versus 35 of 88 (39.7%) in the postintervention period (*P* = .0008). The antipseudomonal antibiotic DOT per 1,000 DFI PD decreased as well: 538.3 in the preintervention period versus 272.9 in the postintervention period (*P* < .0001). Guideline adherence to antipseudomonal antibiotic use recommendations improved in the postintervention group: 41 of 105 (39%) in the preintervention period versus 60 of 88 (68%) in the postintervention period (*P* < 0.0001). Most of this decrease was related to the avoidance of antipseudomonal antibiotics when not recommended.

**Fig. 3.**
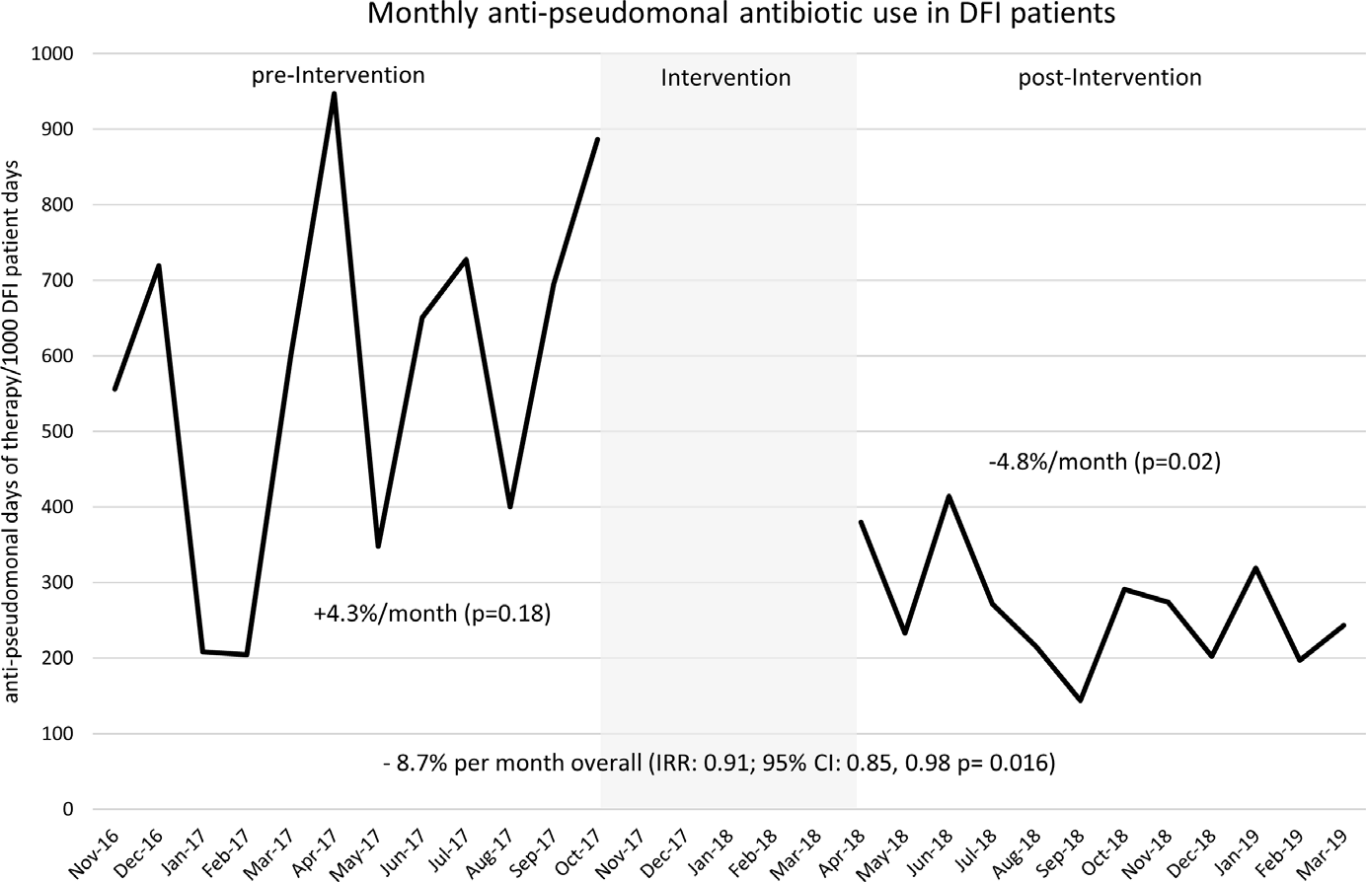
Antipseudomonal days of therapy per 1,000 patient days before and after the intervention. Note. DOT, days of therapy; IRR, incidence rate ratio; CI, confidence interval.

A negative binomial regression model comparing rates of antipseudomonal therapy between the pre- and postintervention periods showed a 54% reduction in empiric antipseudomonal therapy (OR, 0.46; 95% CI, 0.32–0.66). We conducted an interrupted time-series analysis to explore the changes in the level and slope between the 2 periods (Fig. 3). Statistically significant decreases occurred in the level and slope of monthly antipseudomonal therapy after the intervention.

The guideline was downloaded 687 times (409 times in Nebraska and 36 times outside the United States), and the order set was used 41 times during the postintervention period. Order-set usage remained stable, with 49 uses in the 11 months after the postintervention period (April 1, 2019–February 29, 2020).

## Discussion

Our syndromic stewardship intervention was associated with a significant decrease in empiric and overall antipseudomonal antibiotic use in the postintervention period. We targeted antipseudomonal antibiotics because we, and others, had found that *Pseudomonas aeruginosa* was an uncommon DFI pathogen.^13,14^ In comparing the preintervention and postintervention periods, we did not detect differences in important patient-centered outcomes such as mortality, readmission, length of stay, or rates of CDI and amputation. These findings indicate that avoiding empiric antipseudomonal antibiotics in DFI patients admitted to general medical floors is safe when local rates of *Pseudomonas* causing infection are low. Similar interventions targeting skin and soft-tissue infections, and DFI specifically, have yielded reductions in the use of broad-spectrum antibiotics without subsequent increases in adverse clinical outcomes.^9,15^

Our intervention was relatively simple; we utilized 3 components: (1) an evidence-based, locally customized, DFI treatment guideline available on our ASP website; (2) targeted education to providers who frequently manage DFI cases; and (3) implementation of an order set in the EMR.

Our guideline document provided antibiotic recommendations based on infection severity, pathogen-specific risk factors, local microbiologic data, and formulary preferences. Measuring guideline downloads could not be assessed with location specificity beyond the level of the state of Nebraska, but our website has an established reputation at our institution as a source of guidance on treatment of common infections. Access and review of a guideline, however, does not equate to implementation or causation. We were not able to specifically link online utilization to treatment decisions, although no other interventions were performed during the study period to limit antipseudomonal antibiotic use in skin and soft-tissue infections, sepsis, or DFI.

When developing our education, we targeted those who most frequently prescribe antibiotics for DFI at our institution (EM residents, hospitalists, IM/FM residents), and we presented local microbiologic and treatment data in the context of national guidelines to demonstrate opportunities for improvement.

Coupling the guideline and education with a EMR order set also likely contributed to our success. Order sets should be efficiency tools that provide convenient access to best practices.^16^ Our guideline excluded orders not supported by guidelines (ie, swab cultures) while making correct management easy. Along with orders needed for the care of complex diabetic patients (eg, insulin orders and nephrology consultation), we included all items needed for DFI management: support for determining infection severity, prepopulated antibiotic orders, medical and surgical consultations, preferred imaging, vascular studies, etc. The order set was a “one-stop shop” for DFI admissions. The order set was used 41 times during the postintervention period among 88 cases, suggesting that 100% adoption is not necessary for a successful intervention.

We had concern about order-set use degradation over time because much of the education targeted residents and one-third of internal medicine and family medicine residents exit the programs yearly. During the 11 months following our postintervention period (April 2019–February 2020), the order set was utilized an additional 49 times, suggesting durability. Residency training is heavily dependent on practice passed down from senior to junior, and we believe that our practice-improving recommendations have been integrated into this process. Despite our efforts, a substantial portion of patients were not treated in a guideline-adherent manner. This may have occurred for a variety of reasons, including care by providers not included in education (ie, they may not have attended a departmental education event), incomplete penetrance of our education, and other clinical factors.

This study had several limitations. Case selection bias may have been introduced by our retrospective design. This was a single-center study, and our results may not be generalizable to other centers. One potential confounder was that additional education was provided around the beginning of the intervention period when clinicians were recommended to generally avoid vancomycin and piperacillin-tazobactam combination therapy to reduce the risk of kidney injury.^17^ Severe DFI was specifically mentioned as a situation in which combination therapy with vancomycin and piperacillin-tazobactam may be appropriate. During this study, we noted significant decreases in piperacillin-tazobactam use, but we also noted subsequent increases in the use of cefepime. Specific to DFI, we detected an overall decrease in antipseudomonal antibiotic use, suggesting that clinicians changed their spectrum of the therapy rather than replacing one antipseudomonal agent with another. We did not specifically capture the presence of osteomyelitis, although we do not expect the diagnosis of osteomyelitis (which is often not established at the time empiric antibiotics are selected) to have had an effect on empiric antibiotic choices. Additionally, similar LOS and amputation rates between groups does not indicate that major differences in rates of osteomyelitis were present. Other unmeasured factors may also have influenced antibiotic use, although no major changes were made in the activities of the ASP nor were DFIs specifically targeted for review. Although no significant differences were detected in patient-centered outcomes (LOS, mortality, *C. difficile* infection, etc), this study was not powered to detect differences in these outcomes, and these results should be interpreted with caution.

Overall, this pattern of creating a DFI institutional guideline, best-practice order set coupled with targeted education was associated with a safe and significant reduction in antipseudomonal antibiotic use. Our experience suggests that DFI should be recognized as an additional opportunity for syndromic stewardship intervention for hospitals and healthcare systems.

## Supplementary material

For supplementary material accompanying this paper visit http://doi.org/10.1017/ash.2023.123. click here to view supplementary material

## References

[1] The White House national action plan for combating antibiotic-resistant bacteria. Centers for Disease Control and Prevention website. https://www.cdc.gov/drugresistance/us-activities/national-action-plan.html. Published 2015. Accessed February 16, 2022.

[2] Barlam TF , Cosgrove S , Abbo L , et al. Executive summary. Implementing an antibiotic stewardship program: guidelines by the Infectious Diseases Society of America and the Society for Healthcare Epidemiology of America. Clin Infect Dis 2016;62:1197–1202. 27118828 10.1093/cid/ciw217

[3] Pollack LA , Srinivasan A. Core elements of hospital antibiotic stewardship programs from the Centers for Disease Control and Prevention. Clin Infect Dis 2014;59 suppl 3:S97–S100. 10.1093/cid/ciu542 PMC6521960 25261548

[4] Duhon BM , Hand E , Howell C , et al. Retrospective cohort study evaluating the incidence of diabetic foot infections among hospitalized adults with diabetes in the United States from 1996–2010. Am J Infect Control 2016;44:199–202. 26541067 10.1016/j.ajic.2015.09.012 PMC11694572

[5] Lipsky BA , Berendt A , Cornia P , et al. 2012 Infectious Diseases Society of America clinical practice guideline for the diagnosis and treatment of diabetic foot infections. Clin Infect Dis 2012;54:e132–e173. 22619242 10.1093/cid/cis346

[6] Pence LM , Mock CM , Kays MB , et al. Correlation of adherence to the 2012 Infectious Diseases Society of America practice guidelines with patient outcomes in the treatment of diabetic foot infections in an outpatient parenteral antimicrobial programme. Diabet Med 2014;31:1114–1120. 24825001 10.1111/dme.12501

[7] Yun R , Shakowski C , Fish D. Adherence to IDSA Guidelines for the initial treatment of diabetic foot infections in the emergency department (ED). Open Forum Infect Dis 2015;2:1520.

[8] Cahn A , Elishuv O , Olshtain-Pops K. Establishing a multidisciplinary diabetic foot team in a large tertiary hospital: a workshop. Diabetes Metab Res Rev 2014;30:350–353. 24446250 10.1002/dmrr.2527

[9] Sotto A , Richard JL , Combescure C , et al. Beneficial effects of implementing guidelines on microbiology and costs of infected diabetic foot ulcers. Diabetologia 2010;53:2249–2255. 20571753 10.1007/s00125-010-1828-3

[10] Rittmann B , Stevens MP. Clinical decision support systems and their role in antibiotic stewardship: a systematic review. Curr Infect Dis Rep 2019;21:29. 31342180 10.1007/s11908-019-0683-8

[11] McCreery R , Bergman S , VanSchooneveld T. Adherence to practice guidelines for treating diabetic foot infections: an opportunity for syndromic stewardship. Open Forum Infect Dis 2018;5 suppl 1:S536.

[12] Bernal JL , Cummins S , Gasparrini A. Interrupted time-series regression for the evaluation of public health interventions: a tutorial. Int J Epidemiol 2017;46:348–355. 27283160 10.1093/ije/dyw098 PMC5407170

[13] Uckay I , Holy D , Schoni M , et al. How good are clinicians in predicting the presence of *Pseudomonas* spp in diabetic foot infections? A prospective clinical evaluation. Endocrinol Diabetes Metab 2021;4:e00225. 33855224 10.1002/edm2.225 PMC8029573

[14] Young H , Knepper B , Hernandez W , et al. *Pseudomonas aeruginosa*: an uncommon cause of diabetic foot infection. J Am Podiatr Med Assoc 2015;105:125–129. 25815651 10.7547/0003-0538-105.2.125

[15] Jenkins TC , Knepper B , Sabel A , et al. Decreased antibiotic utilization after implementation of a guideline for inpatient cellulitis and cutaneous abscess. Arch Intern Med 2011;171:1072–1079. 21357799 10.1001/archinternmed.2011.29

[16] McGreevey JD 3rd. Order sets in electronic health records: principles of good practice. Chest 2013;143:228–235. 23276846 10.1378/chest.12-0949

[17] Rutter WC , Cox J , Martin C , et al. Nephrotoxicity during vancomycin therapy in combination with piperacillin-tazobactam or cefepime. Antimicrob Agents Chemother 2017;61:e02089–16. 27895019 10.1128/AAC.02089-16 PMC5278703

