# Supplementary material for: Impact of a syndrome-specific antibiotic stewardship intervention on antipseudomonal antibiotic use in inpatient diabetic foot infection management
Source: Antimicrob Steward Healthc Epidemiol. 2023 Mar 2;3(1):e39. doi: 10.1017/ash.2023.123 (PMC10028944; doi:10.1017/ash.2023.123)
Supplement: Supplementary file 1 [file S2732494X23001237sup001.docx]

Supplemental Material

ICD-10 Code Definitions

Diagnosis Codes:

E08 Diabetes mellitus due to underlying condition

E09 Drug or chemical induced diabetes mellitus

E10 Type 1 diabetes mellitus

E11 Type 2 diabetes mellitus

E13 Other specified diabetes mellitus

A48.0 Gas Gangrene

I96 Gangrene, not elsewhere classified

L02 Cutaneous abscess, furuncle and carbuncle

L03 Cellulitis and acute lymphangitis

L97 Non-pressure chronic ulcer of lower limb, not elsewhere classified

M86 Osteomyelitis

Z44 Encounter for fitting and adjustment of external prosthetic device

Z89 Acquired absence of limb

Procedure Codes:

0J Medical or Surgical Procedure, Subcutaneous Tissue or Fascia

0Q - Medical or Surgical Procedure, Lower Bones

0S – Medical or Surgical Procedure, Lower Joints

0Y- Medical or Surgical Procedure, Anatomical Regions, Lower Extremities
